# Multiscale reorganization of the genome following DNA damage facilitates chromosome translocations via nuclear actin polymerization

**DOI:** 10.1038/s41594-022-00893-6

**Published:** 2022-12-23

**Authors:** Jennifer Zagelbaum, Allana Schooley, Junfei Zhao, Benjamin R. Schrank, Elsa Callen, Shan Zha, Max E. Gottesman, André Nussenzweig, Raul Rabadan, Job Dekker, Jean Gautier

**Affiliations:** 1 Institute for Cancer Genetics, Columbia University Vagelos College of Physicians and Surgeons, New York, NY USA; 2 Integrated Program in Cellular, Molecular, and Biomedical Studies, Columbia University Vagelos College of Physicians and Surgeons, New York, NY USA; 3 Department of Systems Biology, University of Massachusetts Medical School, Worcester, MA USA; 4 Department of Systems Biology, Columbia University Vagelos College of Physicians and Surgeons, New York, NY USA; 5 Laboratory of Genome Integrity, National Institutes of Health, Bethesda, MD USA; 6 Department of Pathology and Cell Biology and Department of Pediatrics, Columbia University Vagelos College of Physicians and Surgeons, New York, NY USA; 7 Herbert Irving Comprehensive Cancer Center, Columbia University Vagelos College of Physicians and Surgeons, New York, NY, USA; 8 Department of Biochemistry and Biophysics, Columbia University Vagelos College of Physicians and Surgeons, New York, NY USA; 9 Howard Hughes Medical Institute, Chevy Chase, MD USA; 10 Department of Genetics and Development, Columbia University Vagelos College of Physicians and Surgeons, New York, NY USA; 11 Present Address: The University of Texas MD Anderson Cancer Center, Houston, TX USA

**Keywords:** DNA damage and repair, Nuclear organization, Chromatin structure, Nuclear organization

## Abstract

Nuclear actin-based movements have been shown to orchestrate clustering of DNA double-strand breaks (DSBs) into homology-directed repair domains. Here we describe multiscale three-dimensional genome reorganization following DNA damage and analyze the contribution of the nuclear WASP-ARP2/3-actin pathway toward chromatin topology alterations and pathologic repair. Hi-C analysis reveals genome-wide, DNA damage-induced chromatin compartment flips facilitated by ARP2/3 that enrich for open, A compartments. Damage promotes interactions between DSBs, which in turn facilitate aberrant, actin-dependent intra- and inter-chromosomal rearrangements. Our work establishes that clustering of resected DSBs into repair domains by nuclear actin assembly is coordinated with multiscale alterations in genome architecture that enable homology-directed repair while also increasing nonhomologous end-joining-dependent translocation frequency.

## Abstract (web summary)

Here the authors report a genomic view of 3D chromatin reorganization following DNA damage. Movement of damaged DNA into nuclear domains, which is brought about by nuclear actin, favors error-free damage repair at the expense of rare chromosome rearrangements with oncogenic potential.

## Main

DNA double-strand breaks (DSBs) are one of the most cytotoxic forms of DNA damage and can arise from exogenous genotoxic insults or as DNA intermediates during physiological DNA transactions^1^. DSB detection and accurate repair are coordinated via the DNA damage response to prevent genome instability and carcinogenesis^2^. Mis-repair is a source of genome instability, and scars of aberrant repair are common in cancer genomes, including translocations, insertions and deletions^3–6^. Two classes of repair process DSBs: end-joining mechanisms, which are primarily nonhomologous end-joining (NHEJ), and homology-directed repair (HDR). NHEJ is a mechanistically flexible pathway used for fast and efficient repair in G1 and G2, whereas HDR is an error-free pathway during S and G2 that uses a sister chromatid as a template for repair. Both end-joining and homology-directed mechanisms are implicated in the generation of translocations^7^. Whereas NHEJ DSBs have limited mobility^8^, live-cell imaging of repair foci and mean-square displacement analysis have revealed the more dynamic nature of HDR DSBs^9–11^. Mobile DSBs assemble into subnuclear compartments, functional domains that increase the efficiency of repair^12^. Studies using microscopy- and sequencing-based approaches have shown that HDR DSBs cluster into domains in G1 (ref. 13) and G2 (refs. 8,14–16).

Both actin- and microtubule-mediated mechanical forces have been implicated in repair domain formation^8,12,17^. We have shown that the actin nucleator ARP2/3 and its activator WASP promote clustering of DSBs into HDR domains. These stimulate repair by facilitating DNA end-resection^8^, the initial step of HDR^1^. In turn, resection leads to increased DSB mobility^8^. Thus, HDR domains arise from the coordinated action of actin-dependent forces and repair reactions.

Our understanding of three-dimensional (3D) organization of chromatin has benefited from improvements in live-cell imaging methodologies as well as the development of super-resolution approaches and fluorescence in situ hybridization-coupled microscopy. In addition, ligation-independent and proximity ligation-based methods, such as Hi-C, are providing an unprecedented view of the multiscale organization of the genome^18^. We are starting to unravel connections between DSB repair and mobility, chromatin context, 3D organization and phase separation^19^. Nonetheless, a broader understanding of the multiscale reorganization of the genome following DNA damage and its consequences is still missing.

Besides facilitating repair^8,14,16,20,21^, the impact of DSB clustering on pathologic chromosome rearrangements remains unclear. For example, bringing DSBs into close proximity may increase the propensity for chromosome translocations, one of the leading causes of oncogenesis. It has been shown that clustering of DSBs in yeast promotes rearrangements^21,22^, and translocating DNA breaks in mammalian cells exhibit varying levels of mobility^23,24^.

Here, we evaluated the genome-wide impact of DNA DSBs on chromatin organization. We demonstrate multiscale rearrangements, including widespread damage-induced compartment shifts and DSB clustering, some of which are regulated by nuclear actin polymerization. Finally, we establish that actin-based movement greatly facilitates chromosome translocations.

## Results

### DNA damage induces local and global chromatin reorganization

**Extended Data Fig. 1:**
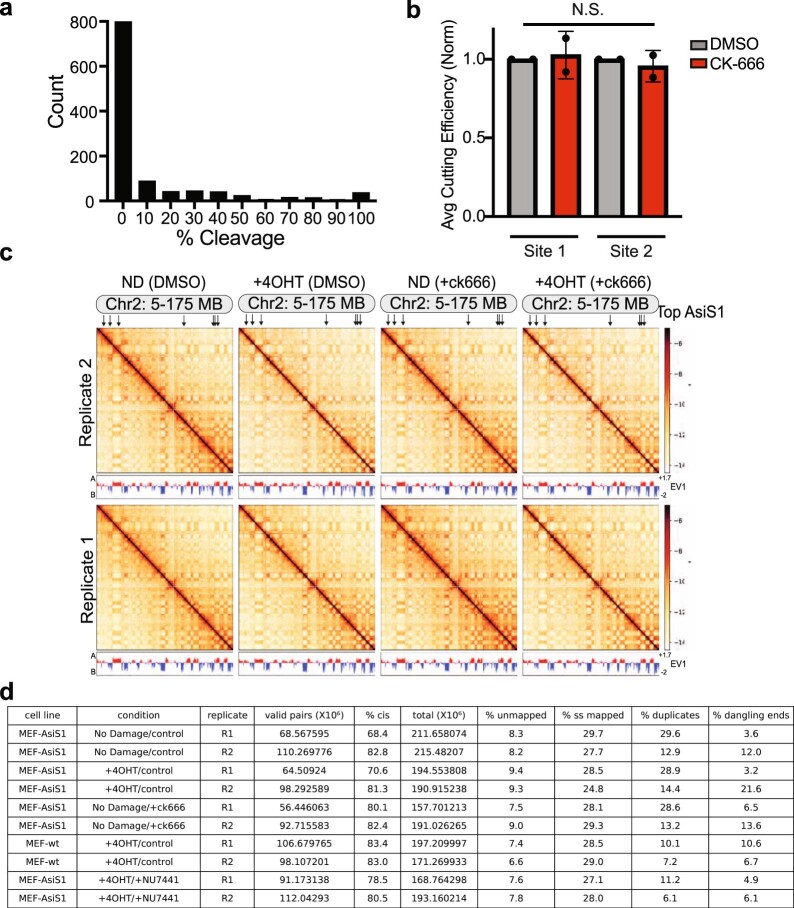
Characterization of AsISI MEFs. **a**, Distribution of AsiSI cutting efficiency for all AsiSI motifs in MEFs as measured by END-seq spike-in assays. **b**, Cutting efficiency of two sites following AsiSI induction (Site 1 = chr2: 13271321, Site 2 = chr3: 88531590) in MEF-AsiSI cells +/− 100 μM CK-666. DNA was extracted from cells 6 hours following damage and % DSBs was measured using quantitative PCR amplification with primers close to the AsiSI sites, normalized to a control (uncleaved) site. Mean and standard deviation. n = 2 biological replicates. **c**, Hi-C interaction frequency maps for a region of chromosome 2 (5–175 Mb) binned at 250 kb and accompanying first eigenvector tracks (EV1) for cis interactions phased by gene density (A > 0). Top 97 frequently digested AsiSI sites in MEFs are indicated by arrows, top. **d**, Hi-C statistics. Source data

DNA damage activates the local, stepwise recruitment of repair proteins to damage sites as well as protein modifications that can spread over megabases along chromatinized DNA, such as the phosphorylation of the histone H2A variant, H2AX (refs. 25–27). Live-cell imaging and chromosome conformation capture-based analyses of DNA repair foci demonstrate that these processes translate into the 3D reorganization of chromatin^8,13^ but do not fully characterize the genomic features of repair domains or the rules that govern their assembly. To assess the impact of DNA damage on genome organization, we performed Hi-C in wild-type mouse embryonic fibroblasts (WT MEFs) as well as MEFs harboring an inducible AsiSI restriction endonuclease (AsiSI-MEFs)^28^. AsiSI-MEFs express AsiSI fused to a truncated estrogen receptor that translocates to the nucleus to trigger DSBs following induction with 4-hydroxy-tamoxifen (4OHT). There are more than 1,000 AsiSI recognition motifs in the mouse genome. However, cleavage efficiency, as measured by END-seq spike-in assays, revealed that the majority of these sites are not cut in MEFs (Extended Data Fig. 1a). Therefore, for a subset of Hi-C data analyses, we focused on the 97 most frequently cut AsiSI sites that showed the highest END-seq signal above background and collectively account for approximately 100 DSBs per cell (Supplementary Table 1)^28,29^. For Hi-C experiments, cells were subjected to DNA damage or treated with dimethylsulfoxide (DMSO) (no damage control) for 6 hours in the presence or absence of additional drugs (Methods). After ensuring that different treatment conditions did not affect the cutting efficiency of AsiSI-ER sites (Extended Data Fig. 1b), two biological replicates of each Hi-C library were performed. Independent analysis of Hi-C experiment replicates showed comparable phenotypes (Extended Data Fig. 1c,d). Therefore, replicates were downsampled to equal read depth and then pooled for analyses presented in the main figures.

**Fig. 1:**
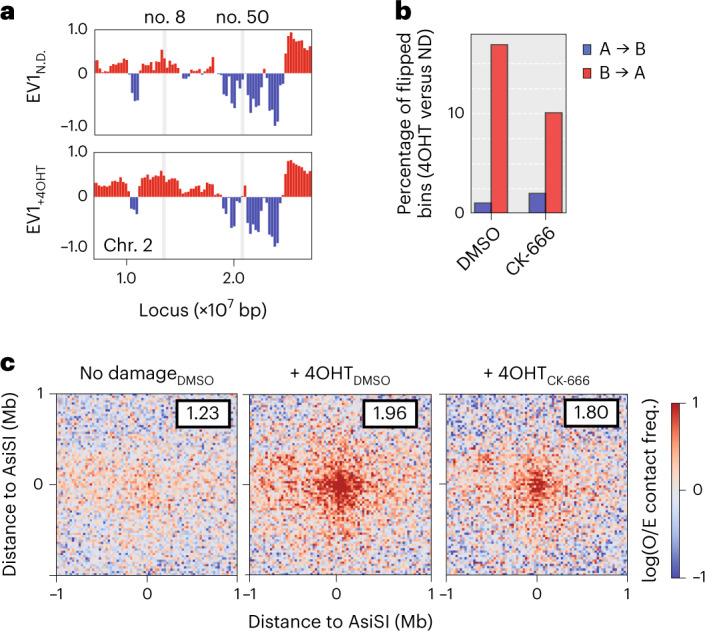
DNA damage induces multiscale alterations of the 3D genome. **a**, Representative trajectories of compartment flipping events on Chr. 2. First EV1 tracks for *cis* interactions in pooled Hi-C replicates binned at 250 kb resolution in the absence (top) or presence (bottom) of DSB induction. Values are phased by gene density (active chromatin/A compartment >0, red; B compartment <0, blue). **b**, Percentage of A (open) or B (closed) compartment bins (250 kb) that flip genome wide on induction of damage with 4OHT. ND, no damage. **c**, Aggregate plots of log_2_(observed**/**expected) contact frequencies for all possible pairwise combinations of the top 97 AsiSI digested sites (Supplementary Table 1) in *cis* (304 interactions between damaged bins). Data are binned at 25 kb and averaged over a 2 Mb flanking window. Average observed/expected (O/E) contact frequency (freq.) heatmaps are shown in undamaged cells and following induction of DSBs (+4OHT) without (DMSO) or with CK-666. Cluster enrichment scores as indicated are calculated using the ratios of the average interaction frequency of the five central bins (125 kb)/average interaction frequency of the outside bins (125 kb–1 Mb). Source data

**Extended Data Fig. 2:**
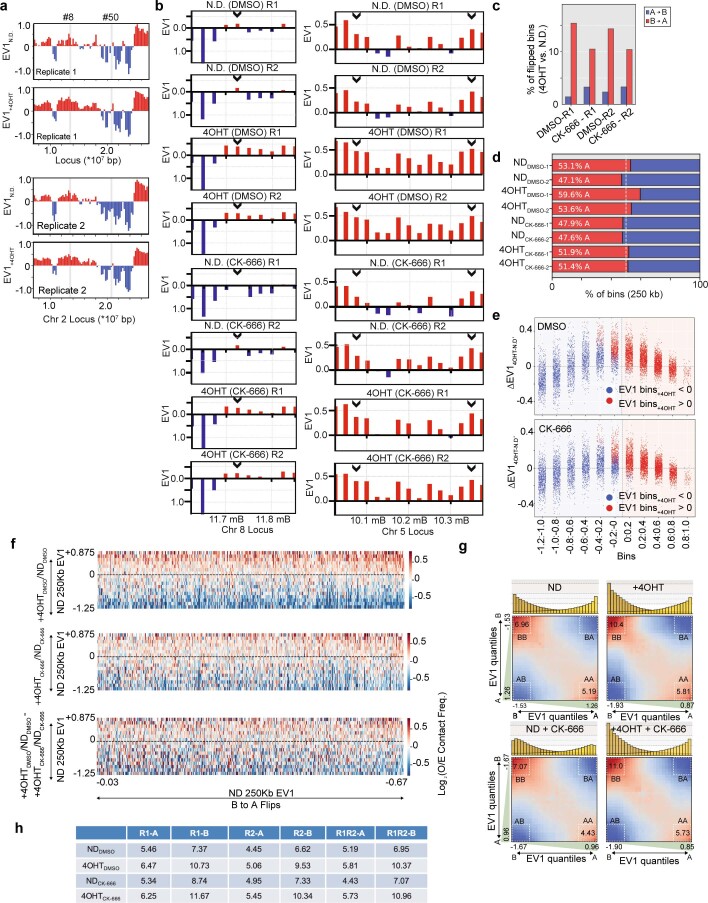
Compartment flips following DNA damage enrich for open (A compartment) chromatin. **a**, **b**, Trajectories of compartment flipping events for individual Hi-C replicates (pooled, Fig. 1a) +/− DNA damage in the presence or absence of ck666. First eigenvector (EV1) tracks for cis interactions at 250 kb resolution before (top) and after (bottom) DSB induction are shown for chromosome 2 (a), 8 (b left), and 5 (b right). Values are phased by gene density (A > 0, red; B < 0, blue). Frequently digested AsISI sites are indicated. **c**, Percent of A (open) or B (closed) compartment bins (250 kb) that flip genome-wide upon DSB induction with 4OHT in individual replicates. Flipped bins are normalized based on A or B compartment status in the matched no damage control. **d**, Fraction of all 250 kb genomic bins assigned to the A (EV1 > 0) or B (EV1 < 0) compartment before and after damage +/− CK-666 (100 μM) in individual replicates. **e**, Genome wide changes in EV1 (+4OHT vs N.D. control) for pooled Hi-C replicates in the absence (DMSO) or presence of ck666 plotted as a function of stratified binned eigenvector values in the matched undamaged control. **f**, Relative Log2 observed/expected contact frequencies (250 kb bins) for pooled Hi-C replicates are binned by EV1 of the matched DMSO control (y-axis) for loci that flip from B to A in both individual replicates +4OHT (x-axis). Flipped loci are stratified on the x-axis by the EV1 of the undamaged DMSO control. Average interaction heatmaps are plotted for the ratio of +4OHT/N.D. interactions in the absence or presence of ck666 (top two panels) as well as the difference between these ratios (DMSO - CK-666, bottom panel). **g**, Saddle plots representing chromatin compartmentalization in pooled Hi-C replicates in the absence or presence of DNA damage (−/+4OHT), −/+ CK-666. The first eigenvector from each library (as indicated) was used to rank 250 kb genomic bins into equal quantiles, excluding the extreme 0.02% of data. The average interaction frequency between these ranked bins was normalized to the expected interactions and used to build the saddle plot. Histograms along the X-axis show the distribution of the A-A and B-B saddle strength, which was quantified by comparing the average interaction frequency of each A-A or B-B quantile bin or bins to the corresponding A-B interactions. Quantification of the preferential A-A and B-B interactions for the top 20% of the strongest A and B loci are indicated. h, Quantification of the preferential A-A and B-B interactions for the top 20% of the strongest A and B loci based on saddle plots for individual replicates. Source data

Hi-C studies have revealed that the genome can be split into A and B compartments that preferentially self-interact and represent open and closed chromatin, respectively^30^. We first sought to examine the impact of DSBs and nuclear actin polymerization on A/B compartmentalization using eigendecomposition of 250-kilobase (kb) binned contact matrices^30,31^, orienting the eigenvector capturing the compartment signal (first eigenvector, EV1) to positively correlate with gene density. Analysis of intrachromosomal interactions following induction of damage revealed a relative increase in EV1, particularly for bins with eigenvector values close to zero (Fig. 1a and Extended Data Fig. 2a–e). On DNA damage (+4OHT), 15% of all bins shifted from B to A compartment identities consistently in both replicates (Fig. 1a,b and Extended Data Fig. 2a–e) and accordingly interacted more frequently with other A loci (EV1 > 0) (Extended Data Fig. 2f). Examples of B to A compartment shifts (blue to red) at or near an AsiSI site are shown (Fig. 1a, DSB no. 50 in Supplementary Table 1 and Extended Data Fig. 2a). Switches were also found at a distance from the AsiSI site, as seen 3.5 Mb (megabases) downstream of DSB no. 8 (Fig. 1a). These compartment changes resulted in genome-wide enrichment of open chromatin following damage, with a consistent increase in the A compartment (Extended Data Fig. 2d). Notably, this A compartment enrichment following DNA damage was dampened by ARP2/3 inhibition with CK-666 (Fig. 1b and Extended Data Fig. 2a–d).

The strength of compartmentalization can be visualized using saddle plots, which display interaction frequencies between pairs of loci arranged according to their EV1 values (Extended Data Fig. 2g,h) and can be quantified to assess the preference for homotypic (A-A, B-B) over heterotypic (A-B) interactions (Extended Data Fig. 2g,h)^32^. Saddle plots for intrachromosomal interactions revealed that DNA damage generally increased homotypic interactions, particularly for the B compartment (Extended Data Fig. 2g,h).

**Extended Data Fig. 3:**
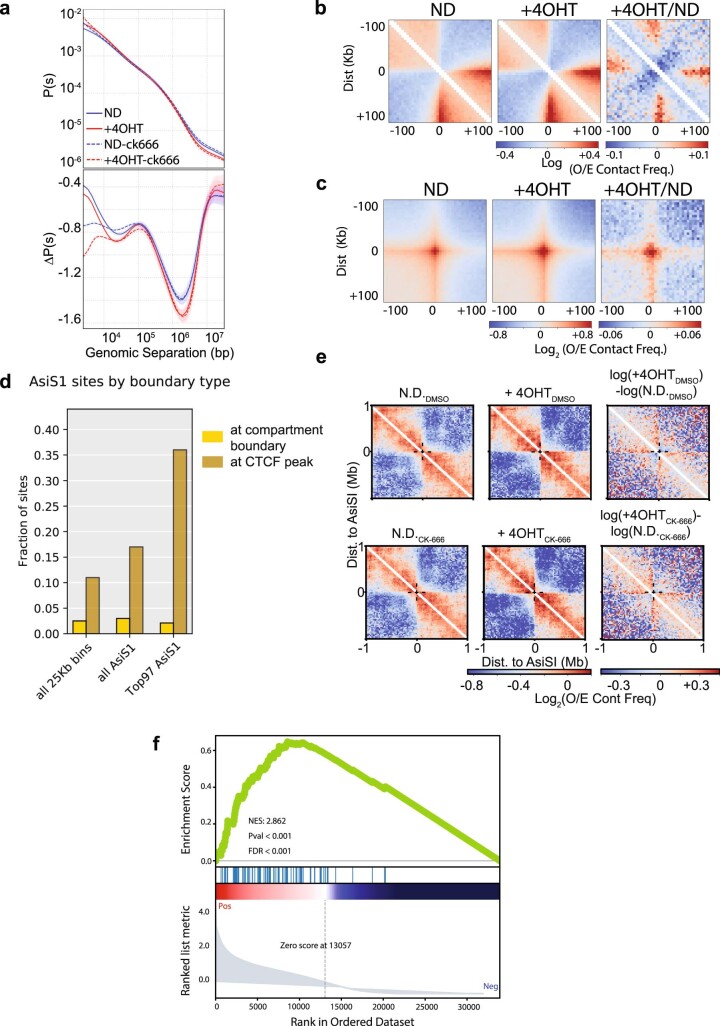
Multiscale genome reorganization following damage. **a**, Top: Contact probability *P* for pooled Hi-C replicates plotted as a function of genomic distance s (*P*(*s*)) for chromosome 2 in the presence or absence of DNA damage (4OHT) +/− CK-666. Bottom: Derivative *P*(*s*) plots. **b**, Average log2(observed/expected) Hi-C interaction frequency maps in the 200 kb regions flanking top CTCF sites (4052) binned at 5 kb resolution (left=no damage, middle + 4OHT, right = ratio +/− damage). **c**, Aggregate plots of log2(observed/expected) Hi-C contact frequencies centered on all possible pairwise combinations of convergent CTCF sites (64044 interactions) binned at 5 kb resolution and averaged for a 200 kb flanking window (left=no damage, middle + 4OHT, right = ratio +/− damage). **d**, Fraction of 25 kb bins overlapping a compartment boundary or CTCF site for the entire genome (left), for bins containing an AsiSI motif (middle), or for bins containing a frequently cut AsiSI motif (right, Supplementary Table 1). **e**, Average log2(observed/expected) Hi-C interaction frequency maps in the 1 Mb regions flanking the most frequently cut AsiSI sites in MEF-AsiSI cells at 25 kb resolution +/− CK-666. **f**, Gene set enrichment analysis (GSEA) plot (score curves) assessing the enrichment of the frequently cut AsiSI sites in transcriptionally active regions. Source data

Contact probability *P* plotted as a function of genomic distance *s* (*P*(*s*)) can reveal properties of chromatin architecture including the size and density of cohesin-dependent loops (Extended Data Fig. 3a)^33^. The derivative of *P*(*s*) typically shows a local peak at *s* of roughly 100 kb, corresponding to the average size of cohesin-mediated loops, followed by a valley at *s* of roughly 2 Mb. The depth of the valley is related to loop density^33^. Analysis of the derivative of *P*(*s*) for Hi-C data obtained from cells harboring DSBs revealed a more pronounced valley at *s* of roughly 2 Mb (Extended Data Fig. 3a, red traces). This suggests a general increase in loop density, consistent with previous observations of increased cohesin recruitment to DSBs, including those induced by restriction endonucleases^34–36^. This could reflect both genome-wide and DSB-localized cohesin-driven loop extrusion, which is further supported by increased line formation in Hi-C data when aggregated at strand-specific CTCF sites (Extended Data Fig. 3b). Such lines in Hi-C data reflect increased cohesin-mediated loop extrusion where one base of the loop is anchored at the CTCF site. Hi-C interaction frequency between pairs of convergent CTCF–CTCF sites also increased on DNA damage (Extended Data Fig. 3c). AsiSI sites, particularly those cleaved frequently, were enriched for CTCF peaks (Extended Data Fig. 3d). These sites were not enriched at compartment boundaries (Extended Data Fig. 3d). The difference in (*P*(*s*)) on DNA damage, as well as average insulation and CTCF loop extrusion, were not diminished in the presence of CK-666 (Extended Data Fig. 3a).

Next, contact matrices spanning 2 Mb around frequently cut AsiSI sites (Supplementary Table 1) were aggregated to visualize interactions in the absence (no damage) and presence (+4OHT) of DSBs (Extended Data Fig. 3e). Average contact maps revealed a striking level of organization in undamaged cells, as seen by strong insulation at the AsiSI motifs (Extended Data Fig. 3e, left). This organization is likely driven by the fact that 35% of AsiSI cleaved sites are at CTCF sites (Extended Data Fig. 3d). Gene set-enrichment analysis, which identifies statistically significant relationships between biological states, showed that frequently cut AsiSI sites are enriched in transcriptionally active areas (Extended Data Fig. 3f). On induction of DSBs, average insulation at these sites increased in AsiSI-MEFs (Extended Data Fig. 3e, middle and right).

**Extended Data Fig. 4:**
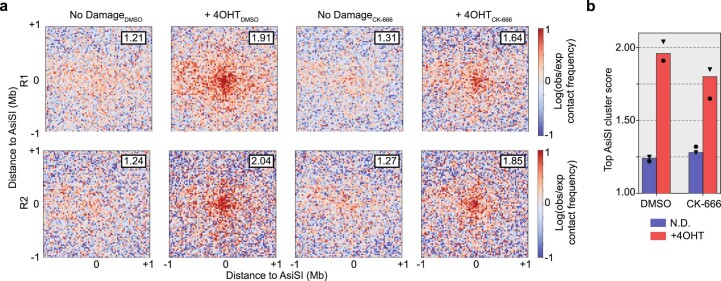
DSB clustering following DNA damage. **a**, Aggregate plots of log2(observed/expected) Hi-C contact frequencies for all possible pairwise combinations of the top 97 AsiSI digested sites (Supplementary Table 1) in cis (304 interactions between damaged bins). Data from individual replicates is binned at 25 kb and averaged for a 2 Mb flanking window. Hi-C maps are made for individual replicate libraries for undamaged or damaged MEF-AsiS1 cells -/+ CK-666. Cluster enrichment scores are calculated using the ratios of the average interaction frequency of the 5 central bins (125 kb) / average interaction frequency of the outside bins (125 kb – 1 Mb) and summarized in b. Cluster enrichment scores are plotted for pooled damaged and undamaged MEF-AsiS1 libraries -/+ CK-666 and individual replicate scores are indicated (R1=circle, R2=triangle). Source data

**Extended Data Fig. 5:**
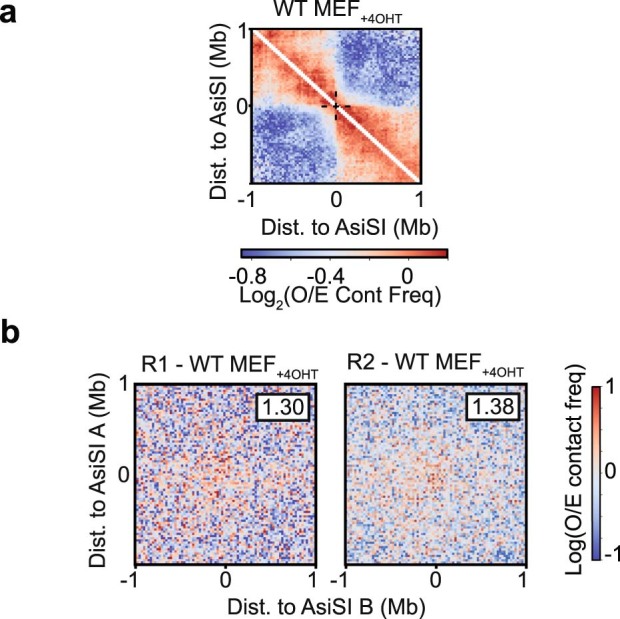
Hi-C analysis in WT-MEF cells. **a**, Average log2(observed/expected) Hi-C interaction frequency for individual replicates maps in the 1 Mb regions flanking the most frequently cut AsiSI sites in WT MEFs without inducible AsiSI-ER at 25 kb resolution. **b**, Aggregate plots of log2(observed/expected) Hi-C contact frequencies for all possible pairwise combinations of the top 97 AsiSI digested sites (Supplementary Table 1) in cis (304 interactions between bins). Data from individual Hi-C replicates is binned at 25 kb and averaged for a 2 Mb flanking window. Cluster enrichment scores as indicated are calculated using the ratios of the average interaction frequency of the 5 central bins (125 kb) / average interaction frequency of the outside bins (125 kb – 1 Mb).

In yeast and mammalian cells, DSB mobility drives clustering of DSBs into repair factories^8,14–16,20,21^. Therefore, we next sought to characterize long-range intrachromosomal interactions between DSBs by aggregating Hi-C data around the 304 possible pairwise interactions in *cis* between the most frequently cut AsiSI sites (Supplementary Table 1)^37,38^. DSB induction in MEF-AsiSI cells triggered DSB clustering (Fig. 1c and Extended Data Fig. 4a,b). Cluster enrichment scores were calculated by comparing the average signal intensity at the center of the plot with that of the surrounding area (Methods), allowing for relative quantification of interaction frequency between AsiSI sites. Clustering increased from 1.23 to 1.96 on addition of 4OHT, indicating that DNA damage triggered increased interactions between distant DSBs within individual chromosomes (Fig. 1c and Extended Data Fig. 4a,b). These distant DSB–DSB interactions increased less on DSB induction when cells were also treated with CK-666 (1.96 to 1.80), suggesting that DNA damage-dependent clustering is driven in part by ARP2/3-dependent nuclear actin polymerization. Insulation at aggregated nuclease-sensitive AsiSI sites (Extended Data Fig. 3e) was also observed in 4OHT-treated WT MEFs lacking the inducible AsiSI-ER transgene, indicating that it was not a direct consequence of 4OHT treatment or due to background AsiSI-ER expression (Extended Data Fig. 5a). At the same time, clustering of cleaved AsiSI sites was not observed in response to 4OHT in WT MEFs (Extended Data Fig. 5b).

Our data reveal genome-wide, multiscale alterations in 3D chromatin organization on DNA damage. We report widespread compartment switching that favors open chromatin states, increased loop extrusion and clustering of distant DSBs following DNA damage, reflecting genome reorganization into subnuclear repair domains. Damage-induced genome reorganization, including A-B compartmentalization and distant DSB clustering are regulated in part by ARP2/3-driven actin polymerization.

### Chromosome translocations occur at sites of DSB clustering

**Fig. 2:**
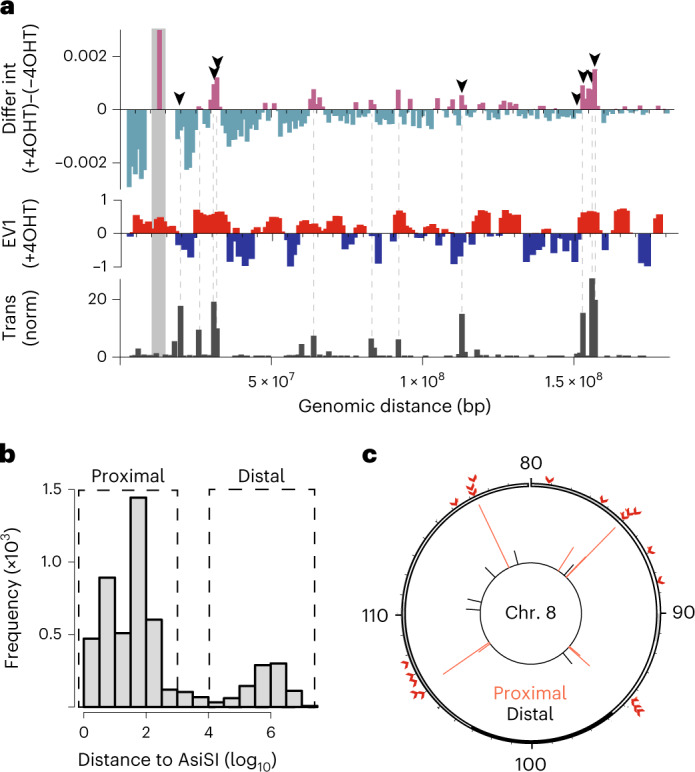
Translocations occur at sites of DSB clustering. **a**, The top shows differential observed/expected Hi-C interactions (4OHT, ND, pooled Hi-C replicates) anchored at the reference site (bait, Chr. 2 13,000,000 Mb) are plotted at 1 Mb resolution. Arrowheads represent frequently cut AsiSI sites. Data adjacent to the bait site along the main diagonal are highlighted in gray (Chr. 2 11,000,000–16,000,000 Mb). Magenta bars indicate increased interactions following damage and teal bars indicate decreased interactions following damage. The middle shows the EV1 track for *cis* interactions (250 kb bins) in the presence of DSBs (+4OHT). Values are phased by gene density (active chromatin/A compartment >0, red; B compartment <0, blue). The bottom shows the normalized translocation frequency (translocations per 1,000 events in the dataset) between reference site (bait) and chromosome 2 loci following damage. **b**, Plot of all translocations as a function of their distance to the nearest AsiSI motif. Data are divided into proximal prey (<500 bp of an AsiSI site) and distal prey (>10 kb from an AsiSI site). **c**, Distribution of translocations within a representative chromosome (Chr. 8, 80–120 Mb). Proximal (recurrent) translocation prey (red lines, inner circle) are located <500 bp of an AsisI site (red arrowheads, outside circle). Distal (spontaneous) translocation prey (black lines, inner circle) are located >10 kb from an AsiSI site.

**Extended Data Fig. 6:**
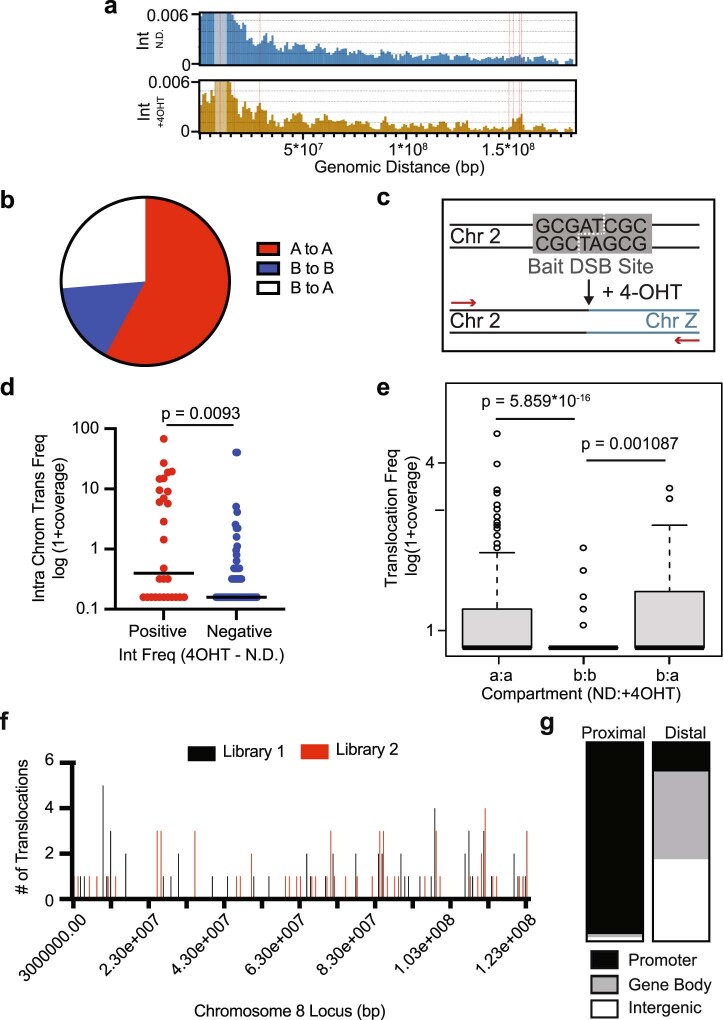
Role of damage-induced genome reorganization in chromosome translocations. **a**, Observed Hi-C interactions in the presence or absence of 4OHT-induced DNA damage (bait, Chr2: 13000000 Mb) and plotted at 1 Mb resolution. Red dotted lines represent frequently cut AsiSI sites. Data adjacent to the bait site along the main diagonal are highlighted in gray (Chr2:11000000-16000000 Mb). **b**, Fraction of bins (1 Mb) that remained in the A compartment (EV1 > 1; red), remained in the B compartment (EV1 < 1; blue), or flipped from the B to the A compartment (white) following DNA damage for a subset of loci on chromosome 2. Analysis includes the 20 bins that displayed the most notable increase in interaction frequency +/− damage. No bins in this subset flipped from compartment A to B. **c**, Schematic of HTGTS experiment. The bait site is located on chromosome 2, 13271321 bp. **d** Normalized translocation frequency from a representative sample plotted for 1 MB bins that had increased interactions following damage (red) or those that had decreased interactions following damage (blue). Bins that did not have any translocations were excluded. P was calculated using Student’s two-tailed t-test. Mean is plotted. **e**, Normalized translocation frequency from a representative sample plotted for 250 kb bins that remained in the A compartment (EV1 > 1; a:a), remained in the B compartment (EV1 < 1; b:b), or flipped from the B to the A compartment (b:a) following DNA damage. Data are shown as box-and-whisker plot in which he box represents the bulk of the data (25th percentile through 75th percentile), whisker lines represent statistical minimum and maximum values, and each dot represents an individual data point. Bins that did not have any translocations were excluded. Median is plotted. P was calculated using Student’s two-tailed t-test. **f**, Distribution of distal prey along chromosome 8 in two individual libraries. **g**, Distribution of proximal (<500 bp from an AsisI site) and distal (>10 kb from an AsiSI site) prey into promoter, gene body, and intergenic categories. Source data

Next, we sought to assess the genetic consequences of these intrachromosomal interactions. We analyzed Hi-C interactions in *cis* anchored at a frequently cleaved AsiSI site on chromosome 2 (reference site DSB no. 8; Supplementary Table 1) and the rest of chromosome 2 at 1 Mb bin resolution (Extended Data Fig. 6a). For differential interaction plots, magenta bars above the axis indicate strengthened interactions following damage, while teal bars below the axis represent a decrease in interaction frequency (Fig. 2a). Concordant with observed DSB clustering in Fig. 1c, the reference cleaved site (gray) preferentially interacted with other cleaved AsiSI sites in *cis* following damage (Fig. 2a, top, arrows), as well as with other genomic loci in the A compartment (Fig. 2a, middle, red bars, and Extended Data Fig. 6b).

We hypothesized that DSB clustering in *cis* during repair domain formation (Fig. 2a) could affect intrachromosomal translocations. Indeed, a substantial fraction of oncogenic translocations take place between loci on the same chromosome^39–41^. Thus, we performed high-throughput genome-wide translocation sequencing (HTGTS) to assess the relationship between damage-induced chromatin reorganization and chromosome rearrangements. HTGTS identifies translocation events between a single fixed ‘bait’ DSB and ‘prey’ sites throughout the genome. We used the AsiSI reference site on chromosome 2 (Fig. 2a, gray bar) as the bait site (Extended Data Fig. 6c)^42^. HTGTS revealed that chromosome 2 loci with increased interactions with the reference site following DNA damage corresponded to sites of frequent translocations with the bait (Fig. 2a, bottom and dashed lines). We then assessed the relationship between contact frequency (+4OHT) and translocation frequency across chromosome 2. Notably, loci with increased interactions following damage exhibited increased translocation frequency compared to loci that had decreased interactions with the bait (Extended Data Fig. 6d). Thus, DNA repair domains are sites where translocations can occur. Furthermore, translocation preys were enriched in the A compartment following damage, including in flipped B:A compartments, indicating that compartment changes during damage-induced genome reorganization may enhance genetic rearrangements (Fig. 2a and Extended Data Fig. 6e).

### ARP2/3-mediated DSB clustering facilitates rearrangements

Given that approximately 100 AsiSI sites are efficiently cut on induction of AsiSI-ER, we anticipated that most recurrent translocations would take place between the AsiSI reference site (bait) and other cleaved AsiSI loci. Indeed, more than 80% of prey originated from within 500 base pairs (bp) of other AsiSI sites (proximal to AsiSI), whereas approximately 15% of translocations occurred 10 kb–100 Mb away from an AsiSI motif (distal to AsISI) (Fig. 2b,c). Translocations between AsiSI-proximal sites and the bait are recurrent, yet not identical to exact duplicates that could result from PCR amplification, as these are filtered out by the HTGTS pipeline. Our data strongly indicate they occur at DSBs with levels of end-resection up to 500 bp. Translocations to distal prey are primarily unique translocations, which are not found between biological replicates (Fig. 2c and Extended Data Fig. 6f). They may arise from spontaneous, physiological DSB lesions forming at sites of intrinsic genome fragility, including R-loops, G4 quadruplexes, stalled replication forks and sites of active transcription^43^. We show in Extended Data Fig. 3f that frequently cleaved AsiSI sites are within transcriptionally active regions. As expected, translocations originating from loci proximal to AsiSI sites were in transcriptionally active areas, such as promoter sequences (Extended Data Fig. 6g). In contrast, translocations originating from regions distal to AsiSI sites occurred throughout the genome and were not enriched at promoters (Extended Data Fig. 6g).

**Fig. 3:**
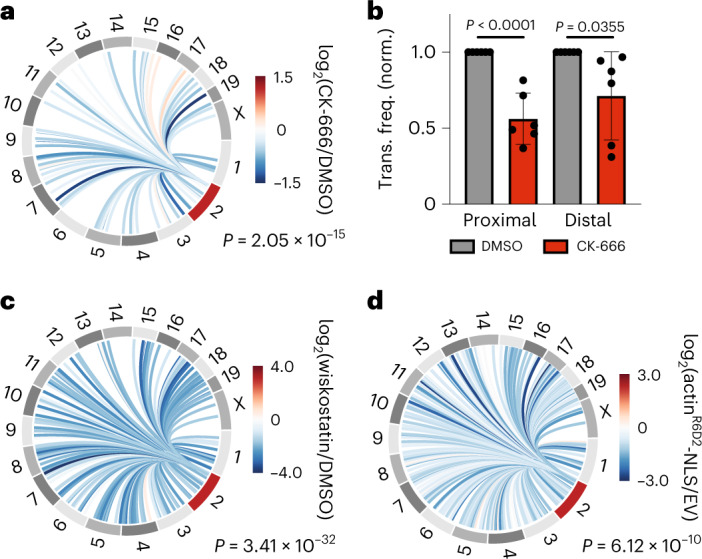
DSB clustering mediated by the WASP-ARP2/3-nuclear actin pathway facilitates chromosome translocations. **a**, One representative Circos plot (out of six biological replicates) visualizing differential normalized translocation frequencies genome wide following damage in the presence or absence of ARP2/3 inhibitor, CK-666 (100 μM) at binned loci that had ≥10 translocation events. Connecting lines are colored according to the log_2_ fold change following damage between ±CK-666 populations. Chromosome 2 (red) contains the bait site. *P* = 2.05 × 10^−15^, Wilcoxon test. Translocations were monitored 6 hours after DSB induction. **b**, Normalized (norm.) translocation frequency (trans. freq.) to proximal (<500 bp of an AsiSI site) and distal (>10 kb from an AsiSI site) prey in MEF-AsiSI cells ±100 μM CK-666. *n* = 6 biological replicates. *P* calculated by Student’s two-tailed *t*-test. Mean is plotted and bars represent the standard deviation. **c**, Circos plot visualizing differential normalized translocation frequencies genome wide following damage in the presence or absence of WASP inhibitor, wiskostatin (3 mM) at binned loci that had ≥10 translocation events. Connecting lines are colored according to the log_2_ fold change following damage between ±wiskostatin populations. Chromosome 2 (red) contains the bait site. Two biological replicates. *P* = 3.41 × 10^−32^, Wilcoxon test. **d**, Circos plot visualizing differential normalized translocation frequencies genome wide following damage in the presence of actin^R62D^-NLS versus empty vector (EV) at binned loci that had ≥10 translocation events. Connecting lines are colored according to the log_2_ fold change following damage between ±actin^R62D^ populations. Two biological replicates. Chromosome 2 (red) contains the bait site. *P* = 6.12 × 10^−10^, Wilcoxon test. Source data

**Extended Data Fig. 7:**
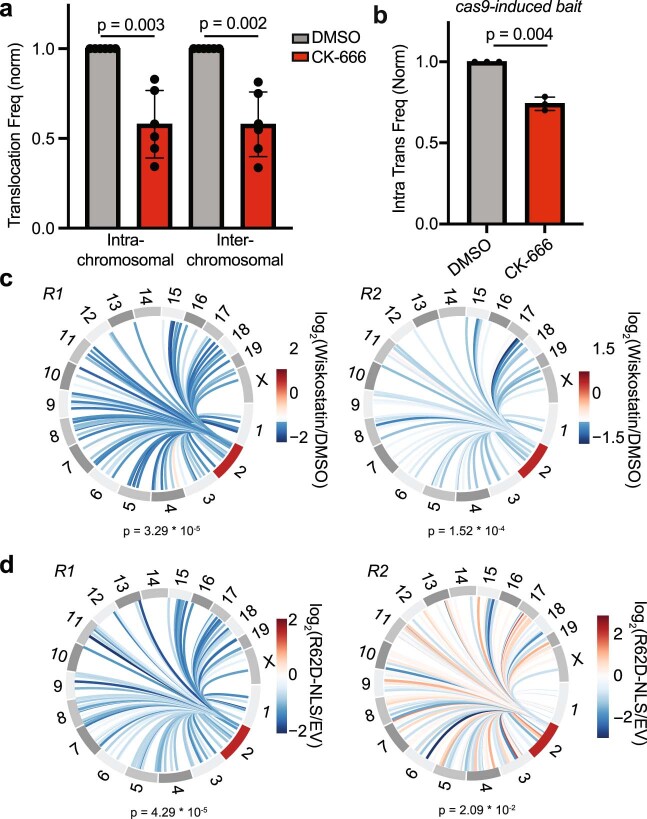
Nuclear-actin-mediated DSB mobility facilitates chromosome translocations. **a**, Normalized translocation frequency to intrachromosomal (chr2) or intrachromosomal prey in MEF AsiSI cells +/− 100 μM CK-666. n = 6 biological replicates. P calculated by Student’s two-tailed t-test. Mean is plotted and bars represent the standard deviation. **b**, Normalized translocation frequency to intrachromosomal prey (chr2) following induction of damage with a cas9-induced bait in WT MEFs (no AsiSI-ER) +/− CK-666. Translocations were assessed 48 hours following transfection with cas9 plasmid containing sgRNA targeting chromosome 2. n = 3 biological replicates. *P* calculated by Student’s two-tailed t-test. Mean is plotted and bars represent the standard deviation. **c**, Circos plots of individual replicates visualizing differential normalized translocation frequencies genome-wide following damage in the presence or absence of WASP inhibitor, wiskostatin (3 μM) at binned loci that had ≥10 translocation events. Connecting lines are colored according to the log2 fold change following damage between +/− wiskostatin populations. Chromosome 2 (red) contains the bait site. P calculated by Wilcoxon test. **d**, Circos plots of individual replicates visualizing differential normalized translocation frequencies genome-wide following damage in the presence of actinR62D-NLS versus empty vector (EV) at binned loci that had ≥10 translocation events. Connecting lines are colored according to the log2 fold change following damage between +/− actinR62D populations. Chromosome 2 (red) contains the bait site. *P* calculated by Wilcoxon test. Source data

ARP2/3 facilitates interactions between DSBs (Fig. 1c) and promotes clustering of repair foci^8^. However, it is not known whether nuclear actin dynamics affect the frequency of chromosome translocations. We hypothesized that increased interactions between DSBs drives chromosome rearrangements. Thus, we used HTGTS to assess the impact of ARP2/3 inhibition on translocations genome wide. Translocations were monitored 6 hours after DSB induction in AsiSI-MEFs in the presence or absence of CK-666. Inhibition of ARP2/3 significantly decreased (*P* = 2.05 × 10^−15^) normalized frequency (Methods) of both intra- and inter-chromosomal translocations (Fig. 3a,b and Extended Data Fig. 7a). We also performed HTGTS experiments in WT MEFs harboring a single cas9-induced break at the same locus on chromosome 2. Similarly, we observed that intrachromosomal translocation frequency was also decreased on addition of CK-666 in WT MEF cells harboring a cas9-induced bait site (Extended Data Fig. 7b).

ARP2/3-mediated actin polymerization and subsequent repair domain formation is dependent on activation by WASP^8^. We therefore assessed the impact of wiskostatin, a WASP inhibitor, on translocation frequency. Wiskostatin led to a significant decrease in chromosome translocations, providing further evidence that the WASP-ARP2/3-actin nucleation pathway is a driving force for chromosome rearrangements (Fig. 3c and Extended Data Fig. 7c). Notably, CK-666 and wiskostatin inhibit polymerization of both cytoplasmic and nuclear actin. To confirm that nuclear actin was mediating translocations, we overexpressed NLS-tagged dominant negative actin^R62D^-NLS and monitored translocations^8^. Indeed, specific inhibition of actin polymerization in the nucleus decreased (*P* = 6.12 × 10^−10^) translocation frequency (Fig. 3d and Extended Data Fig. 7d).

We next asked how DSB mobility affected translocations to spontaneous DNA lesions. As was the case with a cas9-induced bait site, which reports AsiSI-independent translocations (Extended Data Fig. 7b), the frequency of translocations to distal DNA lesions in AsiSI-MEFs was significantly decreased following treatment with ARP2/3 inhibitor, confirming that ARP2/3 does not only facilitate translocations between restriction enzyme-induced DSBs, but also spontaneous lesions in fragile genomic regions (Fig. 3b and Extended Data Fig. 7b).

### DNA-dependent protein kinase catalytic subunit (DNA-PKc) promotes translocations while suppressing clustering

**Fig. 4:**
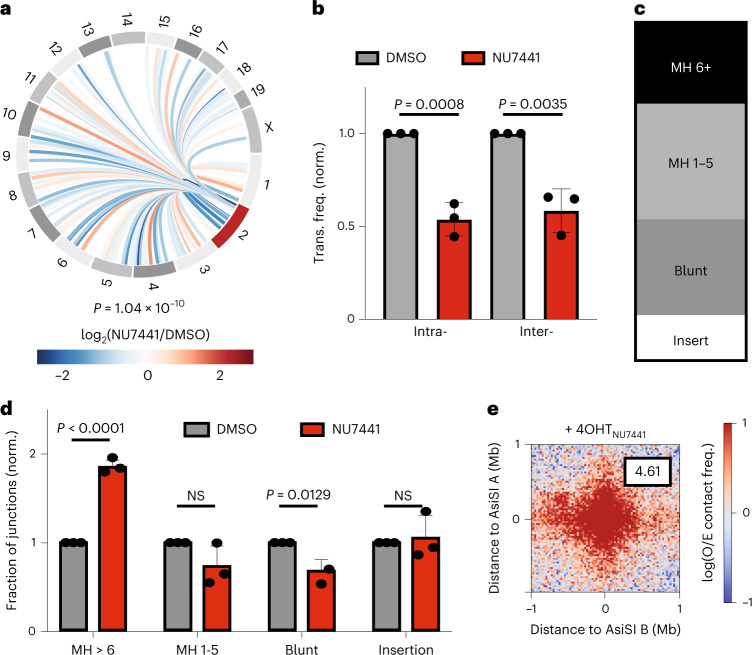
DNA-PKcs promotes translocations while suppressing intrachromosomal interactions. **a**, Circos plot visualizing differential normalized translocation frequencies genome wide following damage in the presence or absence of DNA-PKcs inhibitor, NU7441 (10 mM) at binned loci that had ≥10 translocation events. Connecting lines are colored according to the log_2_ fold change following damage between ±NU7441 populations. Two biological replicates. Chromosome 2 (red) contains the bait site. *P* = 1.04 × 10^−10^, Wilcoxon test. **b**, Normalized (norm.) translocation frequency (trans. freq.) to intra- or inter-chromosomal prey in MEF-AsiSI cells ±0 μM NU441. *n* = 3 biological replicates. *P* calculated by Student’s two-tailed *t*-test. Mean is plotted and bars represent the standard deviation. **c**, Fraction of junctions exhibiting MH >6 nt (MH > 6), MH 1–5 nt (MH 1–5), blunt ends or insertions following DNA damage in one representative sample. **d**, Impact of NU7441 on junctions (normalized to DMSO control) as in **c**. *n* = 3 biological replicates. Mean is plotted and bars represent standard deviation. *P* calculated by Student’s two-tailed *t*-test. NS, not significant. **e**, Aggregate plots of log_2_(observed/expected) contact frequencies for all possible pairwise combinations of the top 97 AsiSI digested sites (Supplementary Table 1) in *cis* (304 interactions between damaged bins) as in Fig. 1 for NU7441-treated (pooled Hi-C replicates), damaged (+4OHT) cells. Cluster enrichment scores as indicated are calculated using the ratios of the average interaction frequency of the five central bins (125 kb)/average interaction frequency of the outside bins (125 kb–1 Mb). Source data

The decrease in translocation frequency on CK-666 exposure strongly suggests that translocations occur within HDR domains assembled from DSBs with resected ends. However, a substantial fraction of translocations generally require NHEJ^44^. Thus, we performed HTGTS in the presence of NU7441, a small molecule inhibitor of DNA-PKcs that selectively compromises NHEJ. As anticipated, NU7441 significantly decreased chromosome translocations, reported by HTGTS at 6 hours, indicating that NHEJ participates in a subset of translocations (Fig. 4a). Nearly 50% of intrachromosomal translocations and 40% of inter-chromosomal translocations were inhibited on NU7441 treatment (Fig. 4b).

DNA sequences at translocation junctions identify repair ‘scars’ and provide further insight into the repair mechanisms driving rearrangements^45,46^. Specifically, the presence of microhomology (MH) suggests repair by alternative end-joining (alt-EJ) whereas blunt-end ligation or very short MHs indicate repair by classical NHEJ (c-NHEJ)^47^. We found that 18% of junctions resulted from blunt-end ligation whereas 69% of junctions harbored MHs (Fig. 4c). As at least 40% of translocations are mediated by NHEJ, this suggests that a fraction of junctions with very short MHs might arise from NHEJ. Consistent with this idea, junctions were significantly enriched in MHs of six nucleotides or more in cells treated with NU7441 (Fig. 4d). In contrast, the relative frequency of blunt junctions was decreased when DNA-PK was inhibited (Fig. 4d).

**Extended Data Fig. 8:**
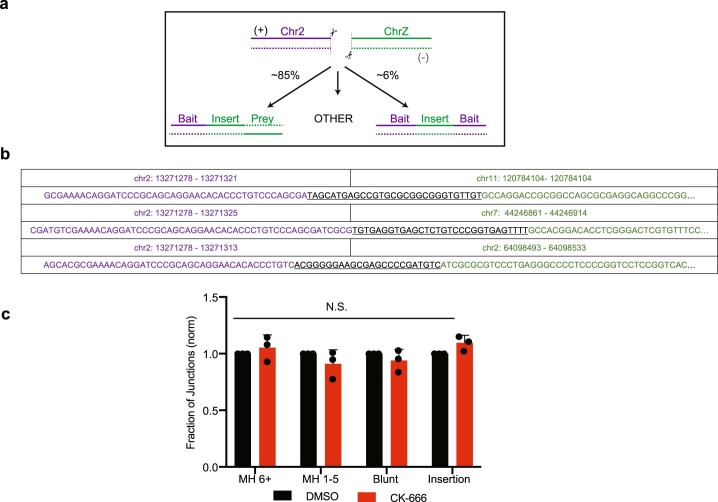
Junctional analysis of translocation events. **a**, Schematic representation of insertion events. **b**, Example reads that contain an insertion event (underline) in between bait (purple) and prey (green). **c**, Impact of CK-666 on fraction of junctions exhibiting microhomology > 6 nt (MH>6), microhomology 1–5 nt (MH 1-5), blunt ends, or insertions following DNA damage. n = 3 biological replicates. Mean is plotted and bars represent standard deviation. Source data

We also observed that 13% of translocations contained additional short insertions. These complex rearrangements did not arise from direct ligation of blunt ends or from annealing of staggered DNA ends between the bait and prey chromosomes (Extended Data Fig. 8a). Of note, HTGTS does not report insertions larger than 30 bp, and therefore insertions may be more frequent than we report. To explore the origins of insertion events, we mapped inserts (20–30 bp). Most inserts (>80%) mapped to the vicinity of prey loci, often on the antiparallel strand (Extended Data Fig. 8b). Insertion analysis suggests that an intermediate step, possibly a transient invasion or annealing event, took place before the ligation that gave rise to a stable translocation. Taken together, these data indicate that alt-EJ and NHEJ both participate in sealing translocation junctions and that breaks, possibly those with longer overhangs, may transiently recombine with proximal sequences.

**Extended Data Fig. 9:**
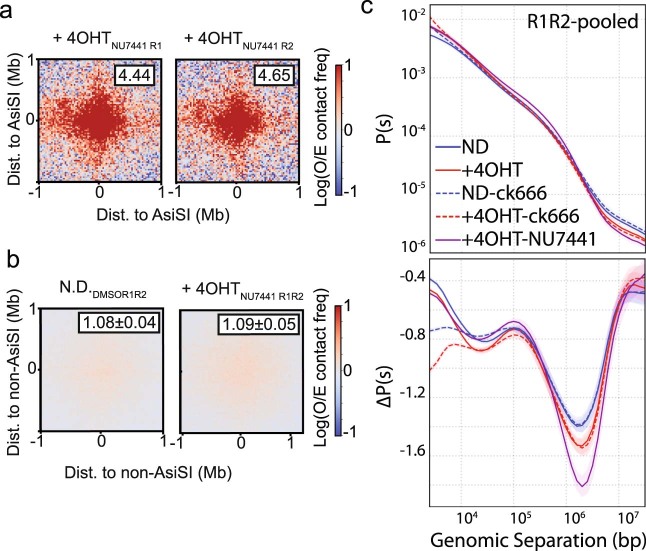
Effect of DNA-PKcs inhibition on damage-induced genome reorganization. **a**, Aggregate plots of Log2(observed/expected) contact frequencies for all possible pairwise combinations of the top 97 AsiSI digested sites (Supplementary Table 1) in cis (304 interactions between damaged bins). Data is binned at 25 kb and averaged over a 2 Mb flanking window. Average observed/expected contact frequency heatmaps are shown for individual Hi-C replicates in the presence of DNA damage and the DNA-PKcs inhibitor, NU7441. Cluster enrichment scores as indicated are calculated using the ratios of the average interaction frequency of the 5 central bins (125 kb) / average interaction frequency of the outside bins (125 kb – 1 Mb). **b**, Aggregate plots of Log2(observed/expected) contact frequencies for all possible pairwise combinations of equivalent (see methods) non-AsiS1 loci in cis (304 interactions between undamaged bins) averaged over 100 random selections for individual Hi-C replicates in the presence or absence of DNA damage +/− DNA-PKcs inhibitor, NU7441. Average cluster enrichment scores as indicated are calculated using the ratios of the average interaction frequency of the 5 central bins (125 kb) / average interaction frequency of the outside bins (125 kb – 1 Mb). The standard deviation from the mean cluster enrichment scores of 100 random non-AsiS1 pile-ups is indicated. **c**, Top: Contact probability *P* plotted as a function of genomic distance s (*P*(*s*)) for chromosome 2 in the presence or absence.

We hypothesized that DNA-PKcs-dependent end-joining acts downstream of movement-dependent clustering of DSBs in the generation of chromosome translocations. If this is the case, DNA-PKcs inhibition should uncouple translocations from clustering and reveal whether stable translocations could contribute to increased DSB clustering observed by Hi-C (Fig. 1c). Hi-C analysis in NU7441-treated cells revealed that average intrachromosomal interaction frequency surrounding pairs of frequently cut AsiS1 sites was dramatically increased following inhibition of DNA-PKcs (Fig. 4e and Extended Data Fig. 9a, compared to Fig. 1c). Notably, NU7441 treatment did not affect looping index, a measure of clustering, of loci distant from DSBs (Extended Data Fig. 9b). Hence, DNA-PKcs-deficient cells exhibited decreased chromosome translocations despite increased DSB clustering (Fig. 4a,e). This indicates that translocations can be uncoupled from DSB clustering and strongly suggests that stable chromosome translocations are not a main contributor to the level of clustering of DSBS observed by Hi-C analysis. In addition to increased clustering, NU4771-treated cells exhibited altered genomic scaling (*P*(*s*)) that could reflect increased loop density (Extended Data Fig. 9c).

These dramatic changes observed on inhibition of DNA-PKcs may in part be due to the accumulation of unrepaired DSBs as a result of defective NHEJ. This could provide additional substrates for DNA end-resection, leading to increased DSB mobility, clustering and HDR.

## Discussion

DNA damage triggers local signaling to facilitate repair reactions at DNA lesions^48^, subsequent checkpoint activation yielding long-range histone posttranslational modifications within insulated genome boundaries^25,36^ and ARP2/3-mediated DSB mobility^8,16^. Together, these events facilitate the formation of HDR domains. Our studies provide insights into the coordinated, multiscale reorganization of the 3D genome leading to the formation of these repair hubs.

First, we observe DNA damage-dependent, genome-wide changes in chromatin compartmentalization, primarily B to A compartment shifts (Fig. 1a,b and Extended Data Fig. 2). Of note, DSB-induced compartment shifts were attenuated on inhibition of ARP2/3, indicating that nuclear actin-dependent movements facilitate damage-dependent alterations in genome architecture beyond the proximity of DNA lesions (>250 kb away) (Fig. 1b and Extended Data Fig. 2b–d). DNA damage also increases B compartment strength (Extended Data Fig. 2g). We also observed genome-wide increase in the frequency of proximal intrachromosomal contacts following DSB induction, around 2 Mb on *P*(*s*) plots (Extended Data Fig. 3a) and evidence of a modest but consistent increase in loop extrusion features at CTCF sites on DNA damage (Extended Data Fig. 3b,c). Notably, inhibition of DNA-PKcs in damaged cells further increased loop density compared to damaged cells treated with DMSO, perhaps reflecting persistent unrepaired damage and subsequent increased resection in the absence of c-NHEJ machinery (Extended Data Fig. 9b). These changes, along with increased insulation around DSBs (Extended Data Fig. 3d), are consistent with previous reports where cohesin accumulates at sites of DNA damage and promotes extrusion within but not between TADs^36,49–51^.

**Fig. 5:**
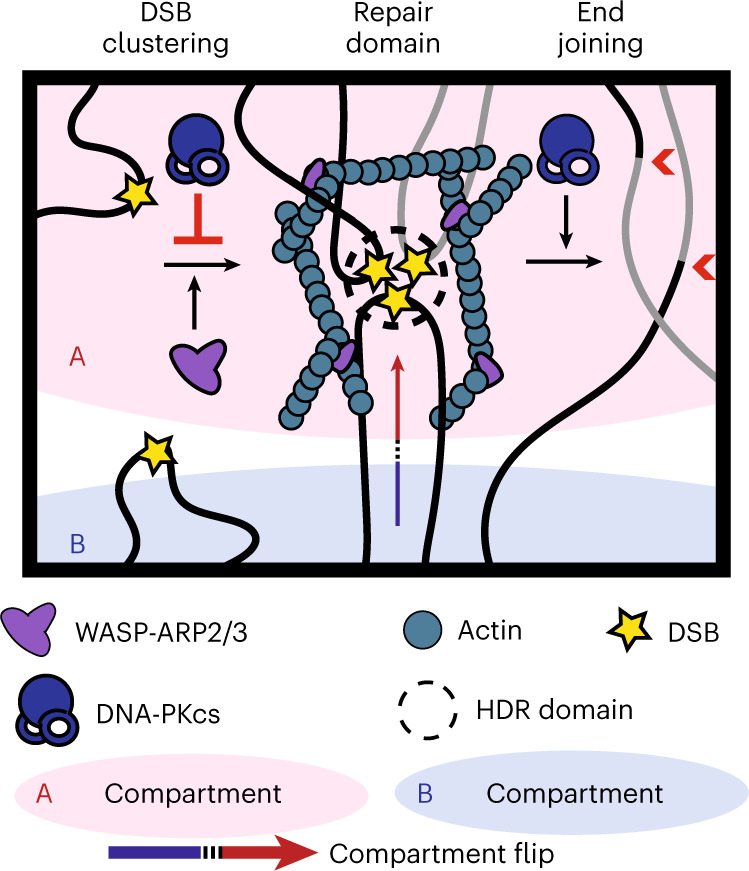
Genome reorganization following DNA damage facilitates translocations. Schematic representation of how multiscale changes in the 3D genome following damage facilitate translocations.

Second, we provide a genomic view of DSB clustering (Figs. 1c and 2a and Extended Data Fig. 4). These data are consistent with a model in which chromatin flips to the A compartment following damage facilitates DSB clustering and the generation of HDR domains (Fig. 5). Notably, while damage-induced, genome-wide chromosome reorganization, such as clustering of DSBs and compartment flips, is facilitated in part by ARP2/3-dependent forces (Fig. 1b,c), local changes in insulation and loop extrusion are not (Extended Data Fig. 3a,e).

Using high-throughput translocation assays (HTGTS), we show that translocations tend to occur in the A compartment (Fig. 2a and Extended Data Fig. 6d) and that WASP-ARP2/3-nuclear actin-mediated clustering of DSBs increases the risk of chromosomal translocations (Fig. 3), while facilitating homologous recombination^8^. We confirm that HTGTS is a powerful method for identifying translocations with naturally unstable loci, establishing that ARP2/3’s impact on chromosomal rearrangements is not limited to restriction endonuclease-generated DSBs but is also relevant for physiological damage. Nevertheless, the incomplete reduction in clustering monitored by Hi-C and in translocations induced by ARP2/3 inhibition points to additional mechanisms for DSB clustering and pathogenic translocations, which may include different actin nucleators^13,52^ or alternative cytoskeleton proteins^16,17^.

Here we establish that clustering of resected DNA ends arising from transcriptionally active loci is also critical for translocations. We also show that chromosome translocations require an end-joining step^53^ (Fig. 4a,b). We thus propose that translocations are generated by a two-step process (Fig. 5). First, actin nucleators (ARP2/3 initiated by WASP) bring DSBs harboring resected ends into close proximity within A compartment (Fig. 5). Second, resected DNA ends are processed to be compatible with end-joining reactions or, alternatively, transiently invade the translocating locus, capturing additional sequences before end-joining (Fig. 4). This is consistent with frequent insertion events observed previously at translocation junctions^54,55^ as well as in this study (Fig. 4c). Overall, our work highlights the delicate balance between faithful repair and mis-repair at play within HDR domains and the critical roles of actin nucleators and repair proteins in achieving this balance.

## Methods

### Cell culture and drug treatment

AsiSI-MEFs were described before^28^. WT MEFs were obtained from R. Baer (Columbia University). MEFs (WT MEFs and AsiSI-MEFs) were cultured in high-glucose Dulbecco’s modified Eagle’s medium supplemented with l-glutamine, 10% fetal bovine serum and 1% penicillin-streptomycin. ER-AsiSI-MEF cell lines were developed as previously described^28^. Cells were treated with doxycycline (Sigma-Aldrich D3072, 3 μg ml^−1^) for 24 h to induce AsiSI expression. 4OHT (Sigma-Aldrich H7904, 1 μg ml^−1^) was added for the last 6 h of doxycycline treatment to induce AsiSI translocation. Cells were cotreated with DMSO, CK-666 (Sigma-Aldrich SML-006, 100 μM), NU7441 (Selleckchem S2638, 10 μM), or wiskostatin (Sigma-Aldrich W2270, 3 μM) and incubated at 37 °C for 6 h. For the DNA-PKc inhibitor experiments, cells were pretreated with 10 μM NU7441 for 1 h before induction of damage with 4OHT. MEF cells were transfected with plasmids using Neon Transfection System (1,350 V, 30 ms, one pulse). For flag-actin experiments, AsiSI-MEF cells were transfected with Flag-actin^R62D^–NLS 24 h before induction of AsiSI expression. For cas9 experiments, WT MEF cells were transfected with single-guide RNA (5′-CCCTGTCCCAGCGATCGCGC-3′) targeting Chr. 2 48 h before cell lysis.

### Hi-C

Chromosome conformation capture experiments were performed as previously described^33^ with some modifications. Briefly, 5 million cells per library were crosslinked with 1% formaldehyde and lysed. After digesting chromatin with 400 units of DpnII overnight, DNA ends were labeled with biotinylated dATP using 50 units Klenow DNA polymerase. Blunt-end ligation was performed with 50 units T4 Ligase at 16 °C for 4 h, followed by reverse crosslinking with 400 μg ml^−1^ proteinase k at 65 °C overnight. DNA was purified using phenol/chloroform extraction and ethanol precipitation, and concentrated on a 30 kDa Amicon Ultra column. Biotin was removed from unligated ends in 50 μl reactions using 50 units of T4 DNA polymerase per 5 mg of DNA. Following DNA sonication (Covaris S220) and Ampure XP size fractionation to generate DNA fragments of 100–300 bp, DNA ends were repaired using 7.5 units of T4 DNA polymerase, 25 units of T4 polynucleotide kinase and 2.5 units of Klenow DNA polymerase. Libraries were enriched for ligation products by biotin pulldown with MyOne streptavidin C1 beads. To prepare for sequencing, A-tailing was performed using 15 units of Klenow DNA polymerase (3′–5′ exo-) and Illumina TruSeq DNA LT Kit Set A indexed adapters were ligated. Libraries were amplified in PCR reactions for 5–7 cycles and subjected to Ampure XP size selection before sequencing on an Illumina HiSeq 4000 machine using the Paired End 50 bp module. Two biological replicates were performed for each condition.

### Hi-C analysis

Paired-end 50-bp reads were processed using the distiller pipeline^56^. First, reads from MEF libraries were mapped to mm10 using BWA-MEM (BWA-0.7.17 (r1188)) in single-sided mode (-SP). Alignments were then parsed, classified and filtered using pairtools^56^. The resulting valid pairs included uniquely mapped and rescued pairs with a minimum mapping quality of 30. To have equal read depth, individual deduplicated replicate libraries were downsampled to 56,446,063 unique valid pairs each, and biological replicates were pooled where indicated for a total of 112,892,126 unique valid pairs. Valid pairs were aggregated into binned contact matrices and kept as multi-resolution cooler files^57^ for subsequent analyses. All Hi-C contact matrices were normalized by iterative correction^31^, excluding the first two diagonals. Downstream analyses were performed using cooltools v.0.3.2 (ref. 58) unless otherwise indicated, python v.3.7.10 and matplotlib version 3.5.2 (ref. 59). Hi-C interaction heatmaps were generated from balanced 250-kb resolution cooler files using cooler ‘show’.

The average contact probability (*P*(*s*)) as a function of genomic distance was calculated using ‘compute-expected’ from cooltools v.0.4.0 (ref. 58). The ‘diagsum’ function was applied to balanced data binned at 1 kb to compute expected, which was then parsed into log-spaced bins of genomic distance using ‘logbin-expected’. The rate of contact frequency decay as genomic distance increases, the *P*(*s*) derivative, was determined using ‘combine_binned_expected’ and provides a highly informative representation of Hi-C data.

Active and inactive chromatin compartments were assigned based on eigenvector decomposition of observed/expected *cis* contact matrices binned at 250 kb resolution using the cooltools ‘call-compartments’ function. In this case, the EV1 was phased according to a positive correlation with gene density with assignment of A (EV1 > 0) or B (EV1 < 0) compartment identity based on high or low gene density, respectively. Saddle plots for *cis* interactions were generated using cooltools ‘compute-saddle’. The ranked 250-kb EV1 values for each library were binned into 30 equal quantiles after excluding the outer 0.2 percentiles of the data. Average observed/expected interactions between bins were then used to build an interaction matrix stratified by these similar EV1 values. The saddle strength is a measure of the relative average observed/expected frequency of interactions within the A or B compartment and can be quantified by taking the average interaction frequency of each A-A or B-B quantile bin or bins divided by the corresponding A-B and B-A bins^32^. Visually, this corresponds to the ratio of the upper left (B-B) or lower right corner (A-A) bins of the saddle plot versus the lower left or upper right corners, respectively. The cumulative ratio for the A (right to left) and B (left to right) compartment, or saddle strength, was plotted above the matrix as indicated in Extended Data Fig. 2e.

Average observed/expected Hi-C interaction frequencies aggregated at subsets of genomic loci were determined using the cooltools ‘snipping’ function. To examine DSB clustering, all pairwise *cis* interactions between bin-aligned top 97-digested AsiS1 sites (Supplementary Table 1) were aggregated at 25 kb resolution with a 2 Mb flanking window. The DSB cluster enrichment score was calculated by taking the ratio of the average Hi-C interaction frequency in the 5 × 5 central bins (25 kb radius) and the average interaction frequency of the remaining bins (125 kb–1 Mb radius). To evaluate the possibility of random clustering, non-AsiS1 loci were selected with the following constraints: (1) undamaged loci had a minimum genomic distance of least 125 kb from any AsiS1 consensus site. (2) The whole chromosome distribution of undamaged loci was equivalent to that of the top 97. (3) The A or B compartment identity (based on EV1 from the undamaged control) was equivalent to that of the top 97. These non-AsiS1 loci were randomly selected with the aforementioned constraints 100 separate times and the average DSB enrichment scores ±s.d. for pairwise *cis* interactions were calculated as described. Loop extrusion features were explored by examining aggregate Hi-C interactions at and around directional CTCF sites. CTCF positions were determined using a previously published CTCF chromatin immunoprecipitation with sequencing dataset from MEFs^60^ (sample GSM2635593). Peaks were called using MACS3 (https://github.com/macs3-project/MACS) with the default ‘callpeak’ parameters and candidate CTCF motifs, generated in HOMER^61^ using a published vertebrate consensus^62^, within 200 bp of these peaks were selected. For pileups, top CTCF sites (13,927 total) were oriented based on the direction of the consensus motif and aggregated at 5 kb bin resolution with a 100 kb flanking window. Loop aggregate plots were generated by considering all possible pairwise combinations of convergent CTCF sites on *cis* chromosomes with a genomic distance of 25–1,000 kb (64,044 possible loops). Loop scores were calculated by taking the ratio of the average Hi-C interaction frequency in the 5 × 5 central bins (25 kb radius) and the average interaction frequency of the remaining bins (25–100 kb radius).

### HTGTS

HTGTS was performed as previously described^42^. Briefly, genomic DNA was collected using phenol/chloroform extraction, sonicated (Covaris S220), and amplified using biotin (5′ Bio-TGGAGAGCGATGAACTGGATC 3′) and nested (5′-NNNNNN***Barcode****CGAAAACAGGATCCCGCAGC*-3′) primers targeting chromosome 2 (chr. 2 13271321). For the nested primer, random nucleotides were added before the barcode to increase library diversity. Sequencing was performed on an Illumina MiSeq sequencer. All primers were purchased from Sigma.

### High-throughput genome-wide translocation analysis

Burrows–Wheeler Aligner was used to align sequences to the mm10 (MEF) genome. Using established pipelines (https://github.com/robinmeyers/transloc_pipeline), reads were filtered with the default parameters. All reads had good mapping quality (mapping quality >30). For translocation frequency, final reads were binned by 100 kb windows genome wide. For each experiment, the number of reads in each window was normalized to the corresponding number of bait-only sequences obtained from the pipeline allowing us to compare translocation frequency between libraries. Genome coordinates of prey sequences were annotated using R package ChIPseeker^63^, which retrieved the location of each prey sequence (Promoter, Gene Body or Intergenic Region). MH analysis was carried out as previously described^46^. MH was defined as the overlapping homologous sequence between the bait and the prey site.

### Quantification of AsiSI-induced DSBs

END-seq experiments and spike-in assays were performed as previously described^28,64^. AsiSI cutting efficiency at specific sites was measured by quantitative PCR^65^ using delta-delta Ct to compare samples ±4OHT. Primers used as follows: site 1 (5′-GTGGGTGTGATTAGGGACCTG-3′, 5′-TGGCCGGATTTTGTGTGC-3′) and site 2 (5′-GACCTGCTCCTCCCACTGTA-3′, 5′-CGGGCCTCTTTCTTATGGTAATGA-3′). Ct was normalized for DNA content using primers distant from any AsiSI motifs (5′-GGACAATGACCGCGTGTTTT-3′, 5′-AACAGCAGGCGCTCTATACC-3′). All primers were purchased from Sigma.

### Gene set-enrichment analysis

GSEAPreranked was used to assess the enrichment of the frequently cut AsiSI sites in transcriptionally active regions. The transcriptional profile of MEF cell line was downloaded from GEO at https://www.ncbi.nlm.nih.gov/geo/query/acc.cgi?acc=GSE29278 (ref. 66) to create the preranked gene list with the level of gene expression as the input. The rank was based on a counts per million value or the number of reads per bin/number of mapped reads (in millions). The null hypothesis is that for ranked bins (counts per million from high to low), AsiSI sites are randomly distributed throughout the list. The closest gene to each AsISI site was collected to make the gene set *.gmt file.

### Reporting summary

Further information on research design is available in the Nature Portfolio Reporting Summary linked to this article.

## Online content

Any methods, additional references, Nature Portfolio reporting summaries, source data, extended data, supplementary information, acknowledgements, peer review information; details of author contributions and competing interests; and statements of data and code availability are available at 10.1038/s41594-022-00893-6.

## Supplementary information

Reporting Summary

Supplementary Table 1 Frequently cut AsiSI sites in MEF cells.

## Data Availability

High-throughput sequencing data have been deposited to Gene Expression Omnibus under accession number GSE183059. Source data are provided with this paper.

## Source data

Source Data Fig. 1 Statistical source data.
Source Data Fig. 3 Statistical source data.
Source Data Fig. 4 Statistical source data.
Source Data Extended Data Fig. 1 Statistical source data.
Source Data Extended Data Fig. 2 Statistical source data.
Source Data Extended Data Fig. 3 Statistical source data.
Source Data Extended Data Fig. 4 Statistical source data.
Source Data Extended Data Fig. 6 Statistical source data.
Source Data Extended Data Fig. 7 Statistical source data.
Source Data Extended Data Fig. 8 Statistical source data.
