# Author Correction: Multiscale reorganization of the genome following DNA damage facilitates chromosome translocations via nuclear actin polymerization

**DOI:** 10.1038/s41594-023-00994-w

**Published:** 2023-04-14

**Authors:** Jennifer Zagelbaum, Allana Schooley, Junfei Zhao, Benjamin R. Schrank, Elsa Callen, Shan Zha, Max E. Gottesman, André Nussenzweig, Raul Rabadan, Job Dekker, Jean Gautier

**Affiliations:** 1 Institute for Cancer Genetics, Columbia University Vagelos College of Physicians and Surgeons, New York, NY USA; 2 Integrated Program in Cellular, Molecular, and Biomedical Studies, Columbia University Vagelos College of Physicians and Surgeons, New York, NY USA; 3 Department of Systems Biology, University of Massachusetts Medical School, Worcester, MA USA; 4 Department of Systems Biology, Columbia University Vagelos College of Physicians and Surgeons, New York, NY USA; 5 Laboratory of Genome Integrity, National Institutes of Health, Bethesda, MD USA; 6 Department of Pathology and Cell Biology and Department of Pediatrics, Columbia University Vagelos College of Physicians and Surgeons, New York, NY USA; 7 Herbert Irving Comprehensive Cancer Center, Columbia University Vagelos College of Physicians and Surgeons, New York, NY, USA; 8 Department of Biochemistry and Biophysics, Columbia University Vagelos College of Physicians and Surgeons, New York, NY USA; 9 Howard Hughes Medical Institute, Chevy Chase, MD USA; 10 Department of Genetics and Development, Columbia University Vagelos College of Physicians and Surgeons, New York, NY USA; 11 Present Address: The University of Texas MD Anderson Cancer Center, Houston, TX USA

**Keywords:** DNA damage and repair, Nuclear organization, Chromatin structure, Nuclear organization

Correction to: *Nature Structural & Molecular Biology* 10.1038/s41594-022-00893-6. Published online 23 December 2022.

This paper was originally published under a standard Springer Nature license (© The Author(s), under exclusive licence to Springer Nature America, Inc.). It is now available as an Open Access paper under a Creative Commons Attribution 4.0 International license, © The Author(s). The error has been corrected in the HTML and PDF versions of the article.

