# Supplementary material for: Multiscale reorganization of the genome following DNA damage facilitates chromosome translocations via nuclear actin polymerization
Source: Nat Struct Mol Biol. 2022 Dec 23;30(1):99–106. doi: 10.1038/s41594-022-00893-6 (PMC10104780; doi:10.1038/s41594-022-00893-6)
Supplement: Supplementary file 1 — Reporting Summary [file 41594_2022_893_MOESM1_ESM.pdf]

nature portfolio

Corresponding author(s): Jennifer Zagelbaum, Allana Schooley, Junfei Zhao, Benjamin R. Schrank, Elsa Callen, Shan Zha, Max E. Gottesman, André Nussenzweig, Raul Rabadan, Job Dekker, and Jean Gautier

Last updated by author(s): Sep 14, 2022

Reporting Summary

Nature Portfolio wishes to improve the reproducibility of the work that we publish. This form provides structure for consistency and transparency in reporting. For further information on Nature Portfolio policies, see our Editorial Policies and the Editorial Policy Checklist.

Statistics

For all statistical analyses, confirm that the following items are present in the figure legend, table legend, main text, or Methods section.

n/a	Confirmed	
☐	☒	The exact sample size (*n*) for each experimental group/condition, given as a discrete number and unit of measurement
☐	☒	A statement on whether measurements were taken from distinct samples or whether the same sample was measured repeatedly
☐	☒	The statistical test(s) used AND whether they are one- or two-sided<br>*Only common tests should be described solely by name; describe more complex techniques in the Methods section.*
☒	☐	A description of all covariates tested
☐	☒	A description of any assumptions or corrections, such as tests of normality and adjustment for multiple comparisons
☐	☒	A full description of the statistical parameters including central tendency (e.g. means) or other basic estimates (e.g. regression coefficient) AND variation (e.g. standard deviation) or associated estimates of uncertainty (e.g. confidence intervals)
☐	☒	For null hypothesis testing, the test statistic (e.g. *F*, *t*, *r*) with confidence intervals, effect sizes, degrees of freedom and *P* value noted<br>*Give P values as exact values whenever suitable.*
☒	☐	For Bayesian analysis, information on the choice of priors and Markov chain Monte Carlo settings
☒	☐	For hierarchical and complex designs, identification of the appropriate level for tests and full reporting of outcomes
☐	☒	Estimates of effect sizes (e.g. Cohen's *d*, Pearson's *r*), indicating how they were calculated

Our web collection on statistics for biologists contains articles on many of the points above.

Software and code

Policy information about availability of computer code

Data collection	Illumina HiSeq 4000 machine (Paired End 50 bp module), Illumina MiSeq sequencer, NIS-elements, MetaCyte software (Metasystems, version 3.10.6)
Data analysis	Data was mapped to mm10 reference genome using BWA-MEM (BWA-0.7.17 (r1188)). For Hi-C analyses, Python 3.7.10, matplotlib, MACS3 (https://github.com/macs3-project/MACS), and HOMER were used. For HTGTS analyses, HTGTS pipeline (https://github.com/robinmeyers/transloc_pipeline), R package ChIPseeker, and GSEAPreranked were used.

For manuscripts utilizing custom algorithms or software that are central to the research but not yet described in published literature, software must be made available to editors and reviewers. We strongly encourage code deposition in a community repository (e.g. GitHub). See the Nature Portfolio guidelines for submitting code & software for further information.

Data

Policy information about availability of data

All manuscripts must include a data availability statement. This statement should provide the following information, where applicable:

- Accession codes, unique identifiers, or web links for publicly available datasets
- A description of any restrictions on data availability
- For clinical datasets or third party data, please ensure that the statement adheres to our policy

High throughput sequencing data have been deposited to Gene Expression Omnibus under accession number GSE183059

Human research participants

Policy information about studies involving human research participants and Sex and Gender in Research.

Reporting on sex and gender	N/A
Population characteristics	N/A
Recruitment	N/A
Ethics oversight	N/A

Note that full information on the approval of the study protocol must also be provided in the manuscript.

Field-specific reporting

Please select the one below that is the best fit for your research. If you are not sure, read the appropriate sections before making your selection.

☒ Life sciences ☐ Behavioural & social sciences ☐ Ecological, evolutionary & environmental sciences

For a reference copy of the document with all sections, see nature.com/documents/nr-reporting-summary-flat.pdf

Life sciences study design

All studies must disclose on these points even when the disclosure is negative.

Sample size	For Hi-C experiments, 2 biological replicates were performed. For all other data, experiments were performed in at least triplicate
Data exclusions	No data was excluded from analysis
Replication	All experiments have at least two independent biological replicates. All findings described in the manuscript were confirmed in all individual replicates.
Randomization	Randomization of this study was not necessary as we did not allocate datasets into experimental groups.
Blinding	Blinding is not required . Results were directly linked with the data and this is not a clinical or genetic study with large numbers of samples.

Reporting for specific materials, systems and methods

We require information from authors about some types of materials, experimental systems and methods used in many studies. Here, indicate whether each material, system or method listed is relevant to your study. If you are not sure if a list item applies to your research, read the appropriate section before selecting a response.

Materials & experimental systems

n/a	Involved in the study	
☒	☐	Antibodies
☐	☒	Eukaryotic cell lines
☒	☐	Palaeontology and archaeology
☒	☐	Animals and other organisms
☒	☐	Clinical data
☒	☐	Dual use research of concern

Methods

n/a	Involved in the study	
☒	☐	ChIP-seq
☒	☐	Flow cytometry
☒	☐	MRI-based neuroimaging

Eukaryotic cell lines

Policy information about cell lines and Sex and Gender in Research

Cell line source(s)	Mouse embryonic fibroblasts
Authentication	Cell lines were authenticated by A. Nussenzweig (MEF)
Mycoplasma contamination	Cells tested negative for mycoplasma

Commonly misidentified lines<br>(See ICLAC register)	N/A
