# Supplementary material for: Multiscale reorganization of the genome following DNA damage facilitates chromosome translocations via nuclear actin polymerization
Source: Nat Struct Mol Biol. 2022 Dec 23;30(1):99–106. doi: 10.1038/s41594-022-00893-6 (PMC10104780; doi:10.1038/s41594-022-00893-6)
Supplement: Supplementary file 2 — Frequently cut AsiSI sites in MEF cells. [file 41594_2022_893_MOESM2_ESM.pdf]

Table 1: Frequently Cut AsiSI Sites in MEF Cells

#	Chromosome	AsiSI Locus
1	15	50869523
2	15	50889504
3	4	84884346
4	4	84884768
5	4	36951817
6	3	88531590
7	5	115632484
8	2	13271321
9	3	38885772
10	8	70283477
11	18	35772093
12	8	106210922
13	2	156144264
14	1	125435720
15	16	20516989
16	1	143739620
17	2	153649707
18	11	53351764
19	8	117256845
20	8	117257071
21	19	57361708
22	7	90443295
23	9	121477514
24	8	84903341
25	8	84952955
26	5	92505504
27	11	94677356
28	15	61986466
29	11	120784167
30	7	44246798
31	11	98203685
32	8	60982849
33	5	73292854
34	2	157135299
35	4	155841144
36	12	111039093

37	8	94666718
38	8	94696176
39	1	89454603
40	2	32236007
41	3	96102864
42	3	96172445
43	11	72607662
44	8	121829355
45	8	36094686
46	8	36094829
47	6	22187041
48	19	5804069
49	9	106247878
50	2	20968934
51	17	74295650
52	2	113847998
53	5	100429798
54	10	85185209
55	1	64121479
56	7	4996048
57	5	129019783
58	5	77265643
59	9	64020793
60	18	84087717
61	8	64849551
62	15	82147584
63	5	88675800
64	6	53287104
65	8	124663162
66	11	103267393
67	11	96777639
68	11	117076963
69	4	139192793
70	5	3596403
71	7	112679954
72	3	9004217
73	6	124662923
74	2	151003957
75	3	60501517

76	3	87906863
77	1	131138137
78	17	86167911
79	4	82505316
80	10	53379805
81	8	127064666
82	2	157424637
83	2	31974705
84	4	155694866
85	19	55743775
86	18	67641762
87	4	119294816
88	7	46795644
89	15	6386610
90	1	133131828
91	X	8192815
92	3	139074691
93	4	46650592
94	X	48594181
95	10	78574874
96	11	102407784
97	11	51619998
